# Transsulfuration Is a Significant Source of Sulfur for Glutathione Production in Human Mammary Epithelial Cells

**DOI:** 10.1155/2013/637897

**Published:** 2013-03-06

**Authors:** Andrea D. Belalcázar, John G. Ball, Leslie M. Frost, Monica A. Valentovic, John Wilkinson

**Affiliations:** ^1^Department of Chemistry, Marshall University, One John Marshall Drive, Huntington, WV 25755-0003, USA; ^2^Department of Pharmacology, Physiology & Toxicology, Marshall University Joan C. Edwards School of Medicine, One John Marshall Drive, Huntington, WV 25755-0003, USA; ^3^Department of Anatomy and Pathology, Marshall University Joan C. Edwards School of Medicine, One John Marshall Drive, Huntington, WV 25755-0003, USA

## Abstract

The transsulfuration pathway, through which homocysteine from the methionine cycle provides sulfur for cystathionine formation, which may subsequently be used for glutathione synthesis, has not heretofore been identified as active in mammary cells. Primary human mammary epithelial cells (HMEC's) were labeled with S35-methionine for 24 hours following pretreatment with a vehicle control, the cysteine biosynthesis inhibitor propargylglycine or the gamma-glutamylcysteine synthesis inhibitor buthionine sulfoximine. Cell lysates were prepared and reacted with glutathione-S-transferase and the fluorescent labeling compound monochlorobimane to form a fluorescent glutathione-bimane conjugate. Comparison of fluorographic and autoradiographic images indicated that glutathione had incorporated S35-methionine demonstrating that functional transsulfuration occurs in mammary cells. Pathway inhibitors reduced incorporation by roughly 80%. Measurement of glutathione production in HMEC's treated with and without hydrogen peroxide and/or pathway inhibitors indicates that the transsulfuration pathway plays a significant role in providing cysteine for glutathione production both normally and under conditions of oxidant stress.

## 1. Introduction

**Figure 1:**
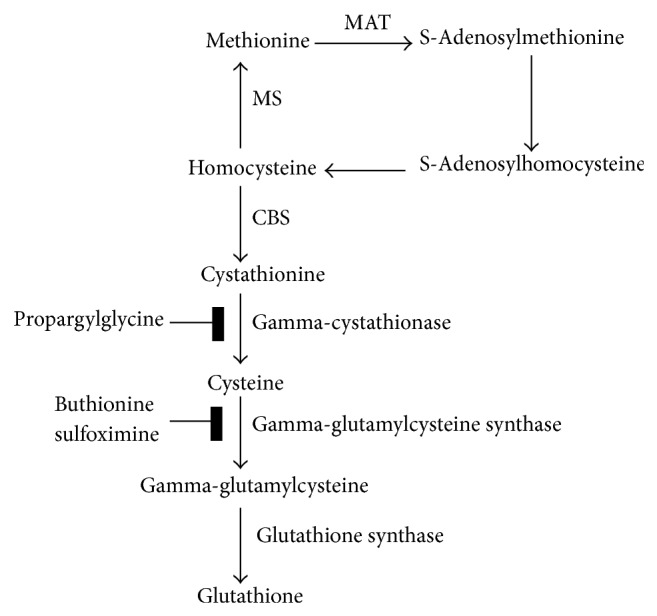
The transsulfuration pathway connects methionine and glutathione biosynthesis. In the methionine cycle, methionine forms S-adenosylmethionine which serves as a methyl donor, generating S-adenosyl homocysteine. This is converted to homocysteine, which is subsequently converted back into methionine. Homocysteine has an alternative fate, however. It can be used to produce cystathionine, which is further converted to cysteine. This latter conversion is catalyzed by gamma-cystathionase and inhibited by propargylglycine. Cysteine can then feed glutathione biosynthesis through production of gamma-glutamylcysteine. This step is catalyzed by gamma-glutamylcysteine synthase and inhibited by buthionine sulfoximine.

In mammals, cystathionine beta-synthase (CBS) catalyzes the first step in the transsulfuration pathway (see Figure 1) [1], a pyridoxal-5′-phosphate- (PLP-) dependent condensation of serine and homocysteine to cystathionine [2, 3]. The second step of the transsulfuration pathway is the hydrolysis of cystathionine to cysteine, ammonia, and *α*-ketobutyrate catalyzed by the enzyme *γ*-cystathionase. These reactions form a metabolic bridge between the methionine cycle and cysteine, a necessary precursor for glutathione biosynthesis.

Homocystinuria is the principal disorder resulting from impairment of transsulfuration (TS) which leads to abnormally high homocysteine levels. Transsulfuration impairment is also associated with other disorders such as autism [4–8], cirrhosis [9–13], coronary artery disease [14–18], goiter [19], impaired coagulation [20–22], immune function [23], neurodegenerative disease [24–26], and pancreatitis [27]. This variety of afflictions highlights the importance of transsulfuration to human health. Despite the widespread nature of tissues involved in these disorders, our knowledge of transsulfuration is confined to the liver and a small number of other tissues. These include the brain [28], certain lymphoid cells [29], and the pancreas [27, 30]. 

Mammary cells may face certain oxidative challenges from diets high in red meat [31–36] (which delivers heme iron [37, 38]) and from ethanol consumption (for which there are many excellent reviews and meta-analyses [39–42]). We are interested in whether transsulfuration occurs in breast tissue to determine its possible role in protection versus oxidant stress for chronic diseases of mammary tissue such as breast cancer. Based on our results obtained using a radioactive tracer method involving ^35^S-methionine incorporation into glutathione, we report that the transsulfuration pathway is active in human mammary epithelial cells. Further work employing pathway inhibitors while measuring glutathione production normally and during oxidant challenge indicates transsulfuration provides necessary sulfur for the enhanced cysteine production that is required for these cells to respond to oxidative stress.

## 2. Materials and Methods

### 2.1. Human Mammary Epithelial Cell Culture

In order to apply the power of a radioactive tracer method to test our hypothesis, while still avoiding as many of the inherent biases that can result from alterations in cellular biochemistry acquired by tissue culture lines as feasible, we used a primary human mammary epithelial cell (HMEC) model system. These cells were obtained from Lonza (Walkersville, MD) and are neither transformed nor immortalized. They are capable of undergoing a limited number of divisions and must be maintained at less than maximum confluence to avoid triggering senescence. Human mammary epithelial cells (HMEC) were grown and passaged under standard conditions in tissue culture treated plasticware (Corning, Lowell, MA) at 37°C at high humidity in water jacketed CO_2_ incubators (Fisher Scientific, Thermo Forma). according to the distributors instructions, changing culture medium every two days with standard medium prepared using their proprietary reagents (Lonza, Walkersville, MD). Standard growth media for HMEC was mammary epithelial basal medium (MEBM), supplemented with growth factors supplied in aliquots at proprietary concentrations, which include (for a 500 mL bottle) 2 mL of bovine pituitary extract, 0.5 mL of epidermal growth factor, 0.5 mL of insulin, 0.5 mL of hydrocortisone, and 0.5 mL of gentamicin sulfate/amphotericin B. To prepare experimental cells, 80% confluent T75 flasks were split and used to seed T75 flasks each with 2E5 cells in 15 mL of standard medium. Seven days after seeding (medium changed every two days), they were 50% confluent and experimental treatment began. 

### 2.2. Transsulfuration Assay

Cells were pretreated for 24 hours with standard media containing either 9 mM buthionine sulfoximine (BSO), 2.5 mM propargylglycine (PPG), or a PBS vehicle control. Then the medium was replaced with 10 mL of radioactive labeling medium (standard media containing the appropriate inhibitors and ^35^S methionine at 2.5 *μ*Ci/mL activity). Cells were incubated for 24 hours, and then pellets were harvested through trypsinization. Pellets were rinsed with PBS and frozen and stored briefly at −80°C.

To fluorescently label cellular glutathione in cell lysates, we adapted the method of Kamencic et al. [43]. Monochlorobimane (mCBi) is reported to specifically react with glutathione (a reaction catalyzed by GST) and not other cellular thiols [44]. Cell pellets were removed from the freezer, an equal volume of water was added, and pellets were thawed and frozen an additional 3 times; following each thaw pellets were vortexed at high speed. The supernatant with cytosolic contents was isolated by spinning the cells at 4°C and 15,000 rpm for 15 minutes. Pellets were reextracted twice, and all supernatants pooled for each sample. The reaction mixture to fluorescently label GSH consisted of 100 *μ*L of cell lysate, 2 *μ*L of mCBi 20 mM, 20 *μ*L of 500 mM potassium phosphate (K_2_PO_4_), pH 6.5 and 2.5 *μ*g of GST (20 *μ*L of 25 *μ*g GST dissolved in 100 *μ*L of PBS) in a total reaction volume of 200 *μ*L (water is used to equalize samples). Reactions were prepared on ice then initiated by incubation at 37°C for a period of 10 minutes. Reactions were stopped by freezing. A control reaction was run by substituting the cell lysate with 2 *μ*L of 10 mM GSH in the same reaction volume of 200 *μ*L. 10 *μ*L of each reaction mixture were spotted on 250 micron silica gel GF uniplates (Analtech, Newark, DE) and analyzed by TLC using a 3 : 1 : 1 mixture of 1-butanol : methanol : water (solvents from Fisher Scientific, Chicago, IL). Two *μ*L of 20 mM mCBi were run in a separate lane as a control to indicate the migration of nonreacted mCBi. Migrations of fluorescent products were compared under UV light and recorded using a Bio-Rad universal hood digital camera (Bio-Rad laboratories, Inc., Segrate, Milan, Italy). Mass spectroscopic analysis confirmed the identity of the GSH-Bimane TLC band (data not shown). Autoradiographs of plates were prepared using Kodak BioMax light film (Sigma, St. Louis, MO) and phosphorimaging using a Typhoon 9200 variable mode imager (Amersham Biosciences, Sweden). Note that the incorporation of methionine into glutathione revealed through autoradiography measures only the glutathione produced from transsulfuration and not glutathione produced from cysteine derived from the media. Further, the incorporation of the fluorescent monochlorobimane label into glutathione occurs regardless of whether the glutathione derives from transsulfuration.

### 2.3. Glutathione Assay

As an initial step in evaluating the biologic significance of active transsulfuration in HMEC's, we hypothesized that the HMEC's would require transsulfuration to produce glutathione in response to oxidant stress. To test this hypothesis, cells were pretreated for 24 hours with vehicle or 2.5 mM PPG inhibitor in standard media. Media was then removed and replaced with media containing pretreatments plus vehicle or 300 *μ*M H_2_O_2_ for two hours. Cells were harvested by trypsinization after the two-hour treatment, with PBS washed pellets frozen in aliquots at −80°C. Cell pellets were homogenized in 500 uL 0.5% sulfosalicylic acid and adjusted to a 1 mL volume. Total glutathione was determined by an enzymatic method [45, 46], as described previously [47–52], using a glutathione reductase and NADPH coupled reaction with 5,5′-dithiobis(2-nitrobenzoic acid) and reported as nmol/g of protein. Protein was determined using the Bradford method [53].

## 3. Results

### 3.1. Transsulfuration Assay

To test our hypothesis that transsulfuration occurs in mammary tissue, we incubated primary human mammary epithelial cells (HEMC's) with a radioactive ^35^S-methionine tracer (Perkin Elmer, Waltham, MA), isolated cellular material following incubation, and determined the extent to which (if any) the ^35^S-methionine incorporated into glutathione. The glutathione in cell extracts was fluorescently labeled with monochlorobimane and separated from other cell constituents using thin layer chromatography. Fluorescent excitation of the TLC plate identified the glutathione spots, while autoradiography of those spots indicated the extent to which ^35^S-methionine incorporated into the glutathione. 

**Figure 2:**
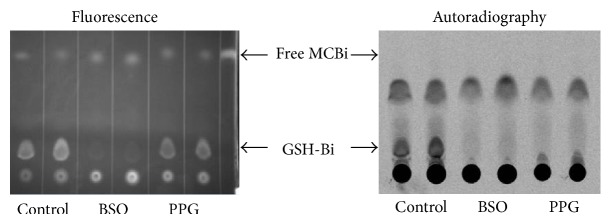
Transsulfuration is a significant source of sulfur for glutathione synthesis in human mammary cells. human mammary epithelial cells (HMEC's) were pretreated with vehicle control, pathway inhibitors buthionine sulfoximine (BSO), or propargylglycine (PPG) for 24 hours then labeled with ^35^S-methionine for 24 hours. Lysate and glutathione bimane conjugates were prepared and analyzed by thin layer chromatography and autoradiography as described under methods. Comparison of the images (fluorescence lanes 1 and 2 with autoradiography lanes 1 and 2) indicates that the fluorescent GSH-MCBi conjugate is radioactive. Incorporation of  ^35^S-methionine into glutathione (GSH-MCBi bands) demonstrates that functional transsulfuration occurs in mammary cells. PPG inhibitory impact on either glutathione synthesis from all cysteine sources (left panel, fluorescence, compare PPG with control) or the incorporation of  ^35^S-methionine labeled cysteine (which must be transsulfuration derived) into glutathione demonstrates both transsulfuration and the identity of the TLC spots. BSO predictably inhibited production of glutathione without regard to cysteine source.

In Figure 2, cell extracts containing bimane-labeled glutathione are analyzed by TLC, and the glutathione conjugates are indicated under the fluorescence panel. Comparison of the images in Figure 2 (fluorescence lanes 1 and 2 with autoradiography lanes 1 and 2) indicate that the fluorescent GSH-MCBi conjugate contains radioactivity. Incorporation of ^35^S-methionine into glutathione (GSH-MCBi bands) demonstrates that functional transsulfuration occurs in mammary cells. The inhibitors propargyl glycine (PPG) or buthionine sulfoximine (BSO) (Sigma, St. Louis, MO) were used to block the pathway at two steps critical for the evaluation of the results (see Figure 1). Cystathionine derives from active transsulfuration wherein homocysteine exits the methionine cycle. Conversion of cystathionine to cysteine is blocked by PPG, while conversion of cysteine to gamma-glutamylcysteine is inhibited by BSO; thus, PPG inhibits incorporation of only transsulfuration derived cysteine into glutathione, while BSO inhibits incorporation of cysteine derived from any source into glutathione [28]. In Figure 2, the impact of PPG inhibition of either total glutathione synthesis (left panel, fluorescence, compare PPG with Control) or the incorporation of ^35^S-methionine into glutathione (right panel, autoradiography, compare PPG with Control) both demonstrate that active transsulfuration is taking place and confirm the identity of the TLC spots. The identity of the spots was further confirmed using mass spectroscopy (data not shown). Pretreatment with BSO caused inhibition of total glutathione (Figure 2: fluorescence, BSO versus control), as well as ^35^S-methionine incorporation (Figure 2: autoradiography, BSO versus control) consistent with its action at one step later in the pathway (Figure 1).

### 3.2. Glutathione Assay

As an initial step in evaluating the biological significance of active transsulfuration in HMEC's, we hypothesized that the HMEC's would require transsulfuration to produce glutathione in response to oxidant stress. To test this hypothesis, cells were pretreated for 24 hours with vehicle or 2.5 mM PPG inhibitor in standard media. Media was then removed and replaced with media containing pretreatments plus vehicle or 300 *μ*M H_2_O_2_ for two hours, and glutathione levels were determined as described under methods. 

**Figure 3:**
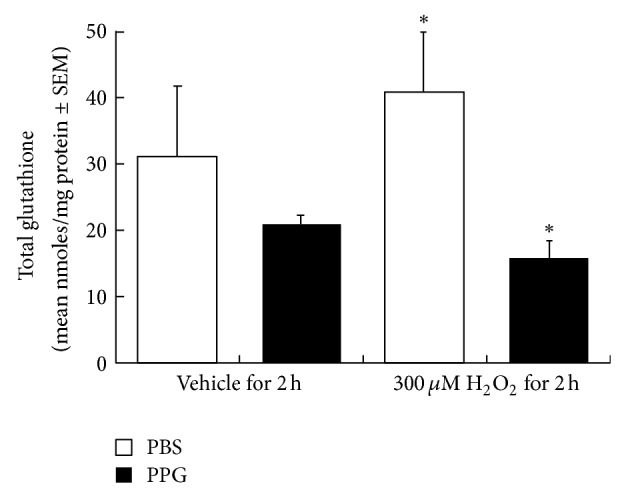
Impact of transsulfuration inhibition (PPG) on cellular total glutathione levels in human mammary epithelial cells subjected to oxidative challenge (H_2_O_2_). Human mammary epithelial cells (HMEC's) were grown in normal mammary epithelial growth medium to 50% confluency and pretreated with a PBS vehicle control or propargylglycine (PPG) for 24 hours, followed by treatment in the same media with vehicle or 300 uM H_2_O_2_ for two hours. Cell pellets were prepared and analyzed for total glutathione levels as described under methods [45]. Results are expressed as nmol/mg of cell protein (mean ± SEM, *N* = 5-6). Asterisk indicates a significant difference between PBS/H_2_O_2_ and PPG/H_2_O_2_ groups determined by ANOVA (*P* < 0.002).

Results, depicted in Figure 3, indicate that resting cells have roughly 30 nmoles glutathione per mg of cell protein. Cells respond to 2 hrs of oxidant treatment by increasing glutathione synthesis to achieve levels of roughly 40 nmoles/mg cell protein. When resting cells are subjected to a transsulfuration blockade using PPG treatment, they contain 20 nmoles GSH/mg cell protein, roughly two-thirds the level of control cells. When cells are oxidant treated while simultaneously undergoing transsulfuration blockade, they are unable to respond to the oxidant insult, resulting in roughly 16 nmoles GSH/mg cell protein. This significantly differs (*P* < 0.002 by ANOVA) from the response in oxidant treated cells which have an active transsulfuration pathway (see Figure 3, compare H_2_O_2_ PBS versus PPG bars). 

## 4. Discussion

Cystathionine *β*-synthase (CBS) catalyzes the first step of the transsulfuration pathway, the conversion of homocysteine to cystathionine, in effect forming a bridge between the methionine cycle and the production of cysteine, a precursor for glutathione biosynthesis. The CBS enzyme is prevalent particularly in the liver and pancreas [30] though its mRNA is also reported to be expressed to a low level in the heart, brain, placenta, lung, skeletal muscle, and kidney [54]. In this same pioneering report, a dot blot did not detect expression for total CBS (representing all 5 known variants of the enzyme) in human mammary mRNA, possibly leading to a lack of further study for this tissue [54]. We speculate that the source of this mammary mRNA must be different than our primary human mammary epithelial cells, which clearly possesses CBS activity, as ^35^S from methionine incorporates into glutathione, seen in Figure 2.

The limitations of our approach, despite our use of nonimmortalized primary cells, involve the artificial nature of an *in vitro* experiment. We chose HMEC's to be able to bring the power of a radioactive tracer approach to answer this question, because generation of ^35^S-glutathione from ^35^S-methionine is the most direct and definitive proof that transsulfuration is active in the cells. Nevertheless, the possibility exists that even carefully tended primary cells will behave differently from cells within tissues, which has the potential to limit the significance of our findings. Our purpose in reporting these *in vitro* results is to progress the field of study in a meaningful way and perhaps provide the basis for subsequent interest in a more involved *in vivo* based work that was beyond our means.

Our results indicate that normal human mammary epithelial cells have an active transsulfuration pathway that significantly contributes to glutathione production. The incorporation of ^35^S-methionine into glutathione, seen in Figure 2, definitively indicates that transsulfuration is active in HMEC's, as the radioactive glutathione can only derive if transsulfuration converts the methionine ultimately to cysteine. Note that mass spectroscopy confirmed the identity of the radioactive, fluorescent band in the thin layer chromatography analysis. The impact of the transsulfuration inhibitor PPG in Figure 3, where total glutathione is measured enzymatically, also indicates that transsulfuration inhibition leads to lower levels of glutathione, implying both the existence of active transsulfuration and an estimate of its contribution to normal glutathione levels. 

When cells are oxidatively challenged, treatment with the transsulfuration inhibitor PPG significantly reduces glutathione levels. While an interesting observation in its own right, these findings imply that oxidant stress in breast tissue may lead to changes in levels of methionine cycle intermediates, such as the methyl donor S-adenosylmethionine, which may result from homocysteine exiting the methionine cycle to replenish glutathione. Because changes in the S-adenosylmethionine methyl donor pool have the potential to impact on DNA methylation, this discovery provides a possible tie between oxidant stress events and the epigenetic regulation of genes involved in disease states. While such a linkage has been reported for liver cells [55], and the epigenetic implications have been discussed [56], our study is the first to explore this area with regard to mammary cells. We feel that the discovery of active transsulfuration in mammary cells is thus a highly significant finding, given that this identifies a metabolic mechanism through which oxidant stress may be linked to the etiology of chronic diseases such as breast cancer, via the potential for changes in the epigenetic regulation of genes. Additionally, the method we employed to analyze transsulfuration provides a straightforward and economical means for such exploration into other tissues.

Because transsulfuration pathway/methionine cycle constituents can be impacted by nutritional interventions that alter vitamin B6 [57], choline, and cysteine [58], or methionine [59], our findings imply that *in vivo* dietary modalities may ultimately be designed to attenuate the potential epigenetic impact of oxidant stress. This may also contribute to our understanding of how diets rich in heme iron, which could be a strong source of dietary oxidant stress, may be linked to breast carcinogenesis. Of great interest, should these potential relationships be demonstrated, would be characterizing the ability of individual polymorphisms of methionine cycle/transsulfuration enzymes to influence this process.
